# Targeting transcription factors associated with hemoglobinopathies: Lessons from successful interventions and implications for cancer

**DOI:** 10.1002/1878-0261.70304

**Published:** 2026-07-16

**Authors:** Meigen Yu, Puspa Das, Xing Zhang, Xiaodong Cheng

**Affiliations:** ^1^ Department of Epigenetics and Molecular Carcinogenesis University of Texas MD Anderson Cancer Center Houston TX USA

**Keywords:** BCL11A, HUDEP‐2, transcription factors, transcriptional circuitry, ZBTB7A

## Abstract

Sickle cell disease and cancer represent fundamentally distinct classes of human disease—one is driven by a defined mutation in β‐globin, whereas the other arises through complex genetic and epigenetic alterations that reshape cellular identity and behavior. Despite these differences, both contexts illustrate how transcription factors, chromatin regulators, and cis‐regulatory elements can impose disease‐relevant gene expression states. In β‐hemoglobinopathies, therapeutic reactivation of fetal hemoglobin through modulation of γ‐globin (*HBG1/2*) regulatory pathways, most notably disruption of the erythroid‐specific BCL11A enhancer, has emerged as a clinically validated strategy. These advances have been facilitated in part by the HUDEP‐2 erythroid progenitor cell line, which provides a tractable adult erythroid model for identifying fetal hemoglobin regulators, validating their function, and evaluating relevant gene editing and gene‐regulatory therapies. Many of the regulators implicated in γ‐globin silencing, including BCL11A, ZBTB7A, NuRD‐associated proteins, DNMT1, KDM1A/LSD1, MYB, and ATF4, also function in cancer‐associated transcriptional or epigenetic networks. In cancer, these factors can support oncogenic transcription, tumor suppressor repression, impaired differentiation, stress adaptation, invasion, or therapy resistance. This review summarizes discoveries enabled by HUDEP‐2 cells in fetal hemoglobin regulation and hemoglobinopathy therapeutic development, then discusses how these mechanisms provide conceptual parallels for understanding and targeting regulatory dependencies in cancer.

## Graphical abstract

This review summarizes the transcription factors, repressive chromatin‐modifying complexes, and epigenetic mechanisms that control fetal hemoglobin repression. Notably, many regulators of γ‐globin silencing also function in transcriptional and epigenetic networks that drive cancer, highlighting opportunities to translate advances in hemoglobinopathy research to oncology. The graph was created by using Microsoft PowerPoint version 16.109.3.

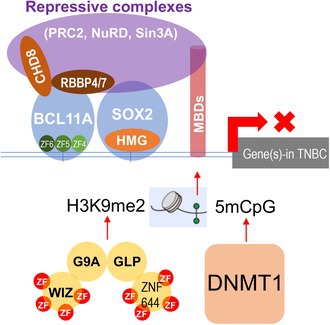

**Abbreviations**
ATF4: activating transcription factor 4
BCL11A: B‐cell lymphoma/leukemia 11A
DNMT1: DNA methyltransferase 1
HBG1/2: gamma‐globin subunits of fetal hemoglobin
HUDEP‐2: human umbilical cord blood‐derived erythroid progenitor cell line
KDM1A: lysine demethylase 1A
LSD1: lysine‐specific demethylase 1
MYB: myeloblastosis proto‐oncogene
NuRD: nucleosome remodeling and deacetylase complex
ZBTB7A: zinc finger and BTB domain‐containing 7A

## 1 Introduction

The study of human erythropoiesis has historically relied on a range of experimental systems, each offering advantages in addition to biological or practical limitations. CD34^+^ hematopoietic stem and progenitor cells (HSPCs) provide a physiologically relevant model but, as donor‐derived primary cells, they are inherently genetically heterogeneous and constrained in scalability and long‐term expansion. The immortalized cell line K562, derived from a patient with chronic myeloid leukemia (CML), has proven valuable for mechanistic studies but represents a transformed, developmentally arrested erythroid state. Consequently, for many years the field lacked a stable and non‐malignant erythroid cell line capable of modeling definitive adult erythropoiesis. This limitation was addressed in 2013 with the establishment of the HUDEP‐2 cell line [1]. HUDEP‐2 cells were derived from umbilical cord blood CD34^+^ HSPCs and conditionally immortalized using doxycycline‐inducible expression of HPV16 E6/E7, permitting sustained expansion while preserving erythroid identity [2]. Importantly, HUDEP‐2 cells predominantly express adult β‐globin, distinguishing them from previously available erythroid models. Since their introduction, HUDEP‐2 cells have been widely adopted for mechanistic studies of globin gene regulation, the development of genome‐editing strategies for the treatment of β‐hemoglobinopathies, and the interrogation of erythroid maturation pathways.

A central feature of human erythropoiesis is the tightly regulated developmental stage‐specific expression of globin genes. Hemoglobin tetramers consist of two α‐like and two β‐like globin subunits whose composition changes over the course of development (Table 1). During embryogenesis, erythroid cells express ζ‐globin (*HBZ*) and ε‐globin (*HBE*). Fetal erythropoiesis is characterized by replacement of ζ‐globin with adult α‐globin (*HBA1* and *HBA2*) and ε‐globin with fetal γ‐globin (*HBG1* and *HBG2*), a process called globin switching. Finally, shortly after birth, γ‐globin expression declines as β‐globin (*HBB*) becomes the dominant β‐like chain, marking the transition from fetal hemoglobin (α_2_γ_2_; HbF) to adult hemoglobin (α_2_β_2_; HbA).

**Table 1:** Expression of α‐ and β‐like globin subunits across developmental stages.

Developmental stage	α‐Globin‐like subunit (chr16)	β‐Globin like subunit (chr11)	SCD mutation	FDA‐approved treatment strategy
Embryonic	ζ‐globin (*HBZ*)	ε‐globin (*HBE*)		
Fetal	α‐globin (*HBA1*, *HBA2*)	γ‐globin (*HBG1, HBG2*)		Increased γ‐globin
Adult	α‐globin (*HBA1*, *HBA2*)	β‐globin (*HBB*)	E6‐to‐V (β‐globin)	

Understanding the developmental regulation of globin genes is clinically relevant for treatment of the β‐hemoglobinopathies sickle cell disease and β‐thalassemia [3]. SCD, first described by Dr. James Herrick in 1910 [4], is caused by the *HBB* c.20A>T (including the first Met) or c.17A>T (not including the first methionine) homozygous point mutation, which results in a Glu‐to‐Val substitution at amino acid 6 position located on the surface of adult β‐globin [5, 6]. The mutant protein produces sickle hemoglobin (α_2_βS_2_; HbS) that polymerizes and leads to erythrocyte sickling [7]. In β‐thalassemia, reduced or absent β‐globin synthesis results in toxic α/β‐globin chain imbalance and ineffective erythropoiesis [8]. Importantly, elevated HbF levels strongly associate with reduced disease severity [9, 10, 11, 12, 13, 14], consistent with a compensatory role for γ‐globin in the setting of defective or absent β‐globin [8, 15, 16, 17]. These observations have motivated extensive study of the molecular mechanisms governing γ‐globin repression and therapeutic strategies aimed at inducing HbF expression. Consequently, experimental models used to study globin regulation must accurately reflect the adult erythroid state in which γ‐globin is developmentally silenced.

Among most popular erythroid cell lines, HUDEP‐2 cells uniquely exhibit a predominantly adult globin profile in which fetal γ‐globin expression is silenced [1]. In contrast, differentiated K562 cells predominantly express embryonic ε‐globin and ζ‐globin, along with substantial fetal γ‐globin [18, 19, 20, 21]. Differentiated CD34^+^ cells, even when derived from adult donors, also retain significant fetal γ‐globin expression in addition to adult α‐ and β‐globin [22]. Consequently, HUDEP‐2 cells provide an important model for dissecting the transcriptional and epigenetic mechanisms that maintain γ‐globin silencing in adult erythroid cells, a critical prerequisite for mechanistic studies of HbF reactivation.

Following the establishment of HUDEP‐2 cells, the use of K562 as a primary model of normal erythroid biology has declined, particularly in studies focused on globin regulation and normal erythroid function. In contrast, CD34^+^ cells remain a critical benchmark system and are frequently used in parallel with HUDEP‐2 cells, particularly for the preclinical evaluation of therapeutics.

The study of human erythropoiesis provides a tractable system for understanding how transcription factors, chromatin regulators, and cis‐regulatory elements establish stable gene expression states. These mechanisms are central not only to normal lineage specification and differentiation, but also to cancer, where aberrant regulatory programs can sustain proliferation, survival, impaired differentiation, stem‐like phenotypes, invasion, and therapy resistance. Accordingly, several regulators first discussed here in the context of γ‐globin repression, including BCL11A, ZBTB7A, and NuRD‐associated proteins, also participate in cancer‐associated transcriptional or epigenetic networks.

Sickle cell disease also provides a clinically validated example of therapeutic gene regulation. In β‐hemoglobinopathies, modulation of fetal hemoglobin expression through perturbation of transcription factors and cis‐regulatory elements has produced meaningful clinical benefit, most notably through disruption of the erythroid‐specific BCL11A enhancer. This therapeutic logic has clear conceptual parallels in oncology, where increasing efforts aim to identify and perturb transcriptional, chromatin‐associated, and regulatory element dependencies that maintain malignant cell states. In this review, we discuss the role of HUDEP‐2 cells in defining mechanisms of fetal hemoglobin regulation and developing hemoglobinopathy‐directed gene therapies, then consider how these regulatory principles intersect with cancer biology and emerging approaches to target oncogenic transcriptional circuitry.

## 2 Research discoveries enabled by HUDEP‐2 cells

Since their establishment, HUDEP‐2 cells have been widely used to investigate the molecular mechanisms governing erythroid biology and globin gene regulation. Specifically, their adult globin expression profile and amenability to precise genetic manipulation have enabled the systematic interrogation of fetal γ‐globin regulators and the development of gene therapy approaches. The following sections highlight key discoveries supported by the HUDEP‐2 model system. Because this section focuses on discoveries enabled by HUDEP‐2 cells, emphasis is placed on studies in which HUDEP‐2 cells were used for functional interrogation or mechanistic validation. More comprehensive, cell‐type agnostic discussions of the individual topics discussed here are available in other dedicated reviews.

### 2.1 Transcriptional regulators of fetal hemoglobin

**Fig. 1:**
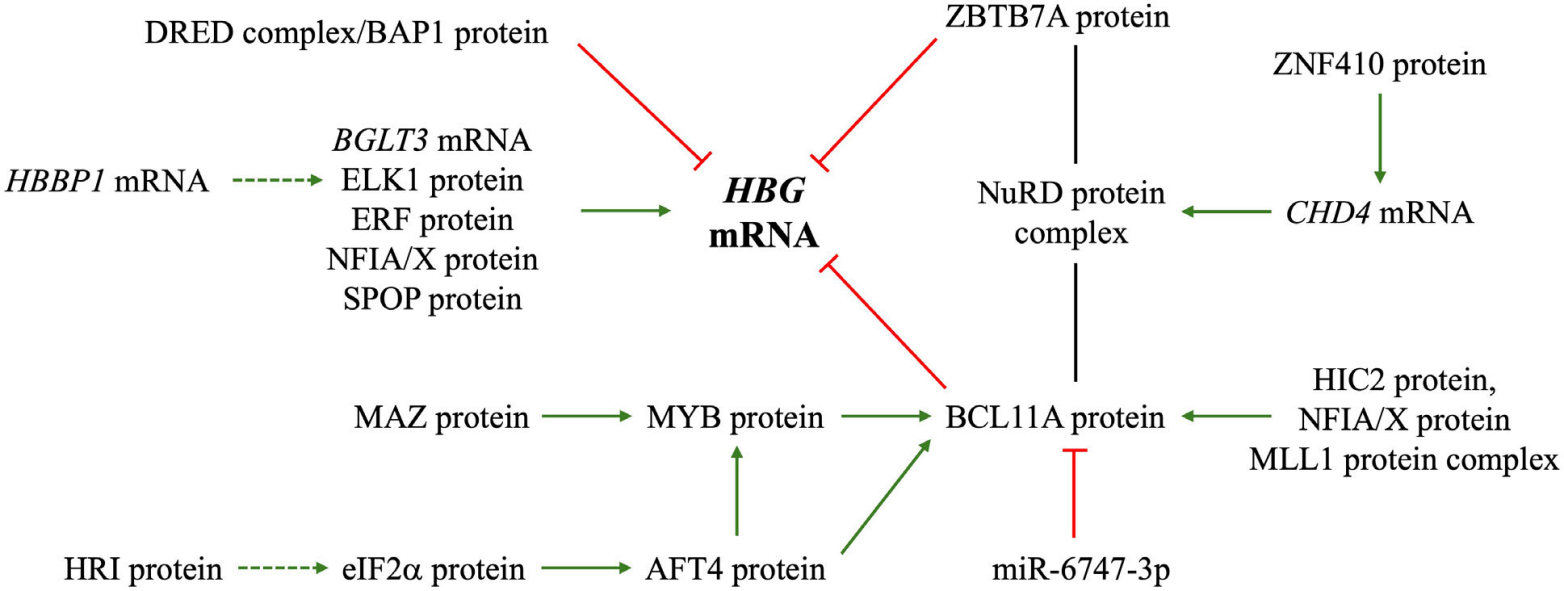
Proteins discovered to regulate fetal hemoglobin (HbF) using HUDEP‐2 cells. Red lines indicate repression. Green arrows indicate activation. Solid lines indicate transcriptional regulation. Dotted lines indicate post‐transcriptional regulation. Graphics were made by using Microsoft PowerPoint version 16.109.3 (Microsoft 365 subscription, License ID: EWW_956a55db‐579d‐42ab‐9f22‐b89fa807e329_c78c83c25f093c274d).

Arguably the most significant impact of HUDEP‐2 cells in erythroid research has been their use in discovering new transcriptional regulators of γ‐globin expression and in defining how established regulators function in adult erythroid cells. The most prominent of these regulators are discussed below. As summarized in Fig. 1, many of the transcriptional regulators of γ‐globin identified in HUDEP‐2 cells, including BCL11A, ZBTB7A, and components of the NuRD complex, converge on shared mechanisms of transcriptional repression.

#### 2.1.1 BCL11A

BCL11A (B‐cell lymphoma/leukemia 11A), a C2H2‐type zinc finger transcription factor, was first implicated in fetal hemoglobin regulation before the establishment of HUDEP‐2 cells, when genome‐wide association studies identified genetic variation at the BCL11A locus as a major determinant of HbF levels [23]. Subsequent studies in primary erythroid cells and other model systems established BCL11A as a functional repressor of fetal γ‐globin expression in adult erythroid cells. Later mechanistic studies in both HUDEP‐2 and CD34^+^ cells have refined this model, showing that BCL11A functions as a tetrameric transcriptional repressor that recruits a silencing complex containing MBD2a (methyl‐CpG‐binding protein 2a)–NuRD and PRMT5 (protein arginine methyltransferase 5) to the *HBG1* and *HBG2* promoters. This complex also excludes transcriptional activators such as the NF‐Y (nuclear factor Y) complex and GATA1 (GATA binding protein 1), thereby enforcing repression through both direct occupancy and competitive binding [24, 25, 26, 27]. These mechanistic findings are consistent with earlier observations in CD34^+^‐derived erythroid cells and other model systems showing that BCL11A associates with the NuRD complex and with erythroid transcription factors including SOX6 (SRY‐box transcription factor 6), GATA1, and ZFPM1 (zinc finger protein, FOG family member 1), and promotes interaction between the β‐globin locus control region (LCR) and the adult β‐globin gene (*HBB*) [28, 29]. High‐resolution chromatin occupancy mapping and systematic single‐nucleotide mutagenesis in HUDEP‐2 cells, together with analysis of naturally occurring mutations associated with elevated HbF levels, further established that BCL11A binding at TGACCA motifs approximately 115 base pairs upstream of the *HBG1* and *HBG2* transcription start sites (−115) is both necessary and sufficient for robust γ‐globin repression [25, 30, 31]. Consistent with this defined binding architecture, recent analysis of BCL11A DNA‐binding specificity demonstrated that BCL11A can also recognize a noncanonical TGCCCA motif, although the functional relevance of these alternative sequences remains unclear [32]. Acute protein degradation studies in HUDEP‐2 cells have additionally identified BCL11A targets beyond the β‐globin locus, with comparison to CD34^+^‐derived erythroblasts indicating that BCL11A‐regulated transcriptional programs only partially overlap between these systems [33, 34].

#### 2.1.2 Regulators of BCL11A

BCL11A expression and activity are subject to multiple layers of regulation in erythroid cells. Numerous transcriptional and post‐transcriptional pathways influencing BCL11A have been described across erythroid model systems, and comprehensive reviews of these regulatory networks are available elsewhere [35].

Among regulators characterized using HUDEP‐2 cells, the transcriptional repressor HIC2 (hypermethylated in cancer 2) has emerged as a notable upstream modulator of BCL11A expression [36]. HIC2 binds to regulatory regions in the BCL11A locus, particularly the +55 and +64 enhancers, suppressing BCL11A transcription by outcompeting the binding of the transcriptional activator GATA1 and reducing enhancer chromatin accessibility. HIC2 expression is highest in fetal erythroid cells and its overexpression partially recapitulates fetal transcriptional signatures, implicating HIC2 as an important stage‐specific regulator in erythroid development. More recently, components of the MLL1 (mixed lineage leukemia‐1) histone methyltransferase complex, a multi‐protein chromatin regulator that catalyzes H3K4 methylation associated with active promoters, were shown to promote BCL11A expression [37]. These factors bind directly to the BCL11A promoter and enhancer, supporting active chromatin states at the locus and reinforcing expression of this major γ‐globin repressor.

BCL11A expression is also influenced by the HRI‐eIF2α‐ATF4 signaling axis. A CRISPR knockdown screen identified the eIF2α kinase HRI (heme‐regulated inhibitor) as a fetal γ‐globin suppressor, demonstrating that loss of HRI increases HbF levels and decreases BCL11A expression [38]. Subsequent studies placed ATF4 (activating transcription factor 4), a transcription factor activated downstream of HRI signaling, as a key mediator of this effect. ATF4 has been shown to directly activate BCL11A transcription via binding to the BCL11A +55 enhancer [39]. Additionally, ATF4 was shown to indirectly regulate BCL11A through binding to the HBS1L (HBS1 like translational GTPase)‐MYB (MYB proto‐oncogene, transcription factor) intergenic enhancer [40], a region in which variants are strongly associated with HbF levels in individuals with β‐hemoglobinopathies [41]. Binding of ATF4 to this region promotes MYB transcription and was proposed as a mechanism by which ATF4 positively regulates BCL11A [40]. However, although MYB is known to suppress HbF expression [42, 43], the extent to which this occurs through regulation of BCL11A remains incompletely defined.

#### 2.1.3 The nucleosome remodeling and deacetylase (NuRD) repressive complex

Silencing of the fetal γ‐globin genes in adult erythroid cells relies on the nucleosome remodeling and deacetylase (NuRD) complex. Genetic perturbation of key NuRD components, including CHD4 (chromodomain helicase DNA‐binding protein 4), MBD2, GATAD2A (GATA zinc finger domain‐containing protein 2A), and HDAC2 (histone deacetylase 2), leads to robust induction of HbF expression, establishing this complex as a central effector of γ‐globin repression [44, 45]. Mechanistically, the MBD2a‐containing NuRD complex occupies the methylated γ‐globin promoters and promotes nucleosome positioning that excludes activating transcription factors, thereby maintaining a transcriptionally repressive chromatin state [26].

As discussed, BCL11A‐mediated recruitment of the NuRD complex represents one major mechanism of γ‐globin repression. Additionally, the transcription factor ZBTB7A (zinc finger and BTB domain‐containing protein 7a; also known as LRF for leukemia/lymphoma‐related factor) also directly represses the γ‐globin genes through recruitment of the NuRD complex [46]. ZBTB7A binds a distinct regulatory element approximately 200 nucleotides upstream of the *HBG* transcription start sites (−200) [46], independent from the −115 TGACCA motif bound by BCL11A [30]. Disruption of ZBTB7A binding results in HbF induction, indicating that BCL11A alone is not sufficient to silence the γ‐globin genes [46]. Consistent with this model, simultaneous reduction of both ZBTB7A and BCL11A produces near‐complete induction of HbF, demonstrating that these factors act through complementary pathways that converge on NuRD‐mediated repression of the γ‐globin locus [46, 47].

In addition to direct repression at the γ‐globin promoters, upstream transcriptional regulators influence silencing by controlling the activity of the NuRD complex itself. The transcription factor ZNF410 (zinc finger protein 410) acts as a highly selective regulator of the NuRD component CHD4 through direct binding to clustered regulatory elements within the CHD4 locus [48, 49]. Depletion of ZNF410 reduces CHD4 expression and weakens NuRD‐dependent repression at the γ‐globin genes, resulting in robust HbF induction with minimal effects on broader erythroid transcriptional programs [45, 48, 49]. This specificity highlights the ZNF410‐CHD4 axis as a potential therapeutic target for pharmacologic HbF induction. In parallel, the NuRD subunit GATAD2A contributes to the formation of the MBD2‐containing repression complex required for γ‐globin silencing, and its loss similarly disrupts NuRD function and elevates HbF expression [50].

#### 2.1.4 Additional transcription factor regulators

In addition to the major transcriptional repressors described above, HUDEP‐2 cells have been used to identify several additional transcription factors that contribute to the regulation of HbF expression. Members of the nuclear factor I (NFI) transcription factor family, NFIA and NFIX, both repress γ‐globin expression by stimulating transcription of BCL11A and by directly binding regulatory elements within the *HBG* locus [51]. The ETS family transcription factor ERF (ETS2 repressor factor) has also been shown to repress γ‐globin expression by binding regulatory regions surrounding the *HBG* genes, suppressing their transcription. ERF knockout does not impair erythroid differentiation, suggesting that ERF controls a regulatory pathway that may be relatively specific to globin gene regulation [52]. The zinc finger transcription factor MAZ (MYC‐associated zinc finger protein) binds directly to the MYB promoter, driving MYB expression and subsequent γ‐globin silencing [53]. This finding adds another layer to the MYB‐HBG regulatory axis, in addition to the HRI pathway discussed earlier, although the potential interplay between ATF4 enhancer‐mediated and MAZ promoter‐mediated regulation of MYB remains to be determined.

#### 2.1.5 Non‐coding RNA regulators

In addition to transcription factors, non‐coding RNAs located within the β‐globin locus have emerged as regulators of γ‐globin expression. Expression of the β‐globin pseudogene *HBBP1* (hemoglobin subunit beta pseudogene 1), located downstream of the *HBG* genes, promotes binding of the transcriptional activator ELK1 (ETS transcription factor ELK1) to the *HBG* promoters and increases γ‐globin transcription [54]. *BGLT3* (beta globin locus transcript 3), a long non‐coding RNA located adjacent to *HBBP1*, can act as an active enhancer of HbF expression. HIF‐1α (hypoxia inducible factor 1 subunit alpha) binding recruits transcriptional co‐activators and increases enhancer‐associated chromatin marks at the *BGLT3* locus, while promoting long‐range interactions between the super‐enhancer β‐globin locus control region and the γ‐globin genes [55]. Interestingly, expression of the *BGLT3* transcript also appears necessary for the pharmacologic induction of HbF expression. Multiple HbF‐inducing compounds – including hydroxyurea, the G9a inhibitor RK‐701, the DNA hypomethylating agent decitabine, and the histone deacetylase (HDAC) inhibitors MS‐275 and SAHA – increase *BGLT3* expression, and depletion of *BGLT3* prevents γ‐globin induction by these compounds [56]. MicroRNAs have also been implicated in this regulatory landscape through modulation of upstream repressors; for example, miR‐6747‐3p directly targets the BCL11A 3′ untranslated region, reducing BCL11A expression and increasing γ‐globin levels [57]. Together, these findings support the importance of non‐coding RNAs in the regulation of γ‐globin expression.

#### 2.1.6 Other regulators

Additional regulators of γ‐globin expression have been identified that do not fall within the major transcription factor or chromatin remodeling pathways described above but nevertheless contribute to repression through distinct or less well‐defined mechanisms. The deubiquitinase BAP1 (BRCA1‐associated deubiquitinase 1) associates with the DRED (direct repeat erythroid‐definitive) repressive protein complex, a heterodimer composed of TR2/NR2C1 (nuclear receptor subfamily 2 group C member 1) and TR4/NR2C2 (nuclear receptor subfamily 2 group C member 2) and the corepressor enzymes DNMT1 (DNA methyltransferase 1) and LSD1 (lysine‐specific demethylase 1). BAP1 stabilizes the assembled DRED complex at the *HBE* and *HBG* gene promoters, promoting γ‐globin repression [58]. Loss of SPOP (speckle type BTB/POZ protein), a substrate adaptor for the CUL3 (cullin 3) E3 ubiquitin ligase complex, induces significant γ‐globin expression independently of BCL11A and ZBTB7A. These findings suggest that SPOP promotes degradation of proteins involved in γ‐globin repression, although the identities of these proteins remain unknown [59].

### 2.2 Genetic mechanisms of fetal hemoglobin (HbF) regulation

Many important insights into the regulation of γ‐globin expression have emerged from studies of genetic variation associated with HbF levels. One source of such insights is the study of mutations causing hereditary persistence of HbF (HPFH), a benign condition characterized by elevated levels of HbF that can alleviate symptoms of SCD and β‐thalassemia. Additional mechanisms regulating γ‐globin expression have been identified through genome‐wide association studies. More recently, advances in genome editing technologies, together with the development of HUDEP‐2 erythroid cell models, have enabled systematic interrogation of both cis‐ and trans‐regulatory elements that contribute to HbF silencing.

#### 2.2.1 Transcription factor bindings sites in the HBG promoters

Studies of HPFH single‐nucleotide polymorphisms (SNPs) have shown that γ‐globin expression is highly sensitive to small changes within the *HBG* promoters. Some HPFH variants disrupt binding sites for transcriptional repressors, such as SNPs within the −115 BCL11A binding site and the −200 ZBTB7A binding site upstream of the *HBG* transcription start site. Importantly, the study of these mutations contributed to the identification of BCL11A and ZBTB7A as direct repressors of γ‐globin expression [47]. Other HPFH mutations increase γ‐globin expression by generating *de novo* binding sites for transcriptional activators. For example, the –113A>G mutation creates a binding site for GATA1 (GATA binding protein 1) [31], while the –198T>C mutation generates a KLF1 (KLF transcription factor 1) binding site [60]. Finally, systematic base editing of the *HBG* promoter regions has identified that the HPFH‐like point mutations −123T>C and −124T>C generate another novel KLF1 binding site capable of inducing HbF expression [61]. Notably, the discussed mutations that generate *de novo* transcriptional activator binding sites occur within or near the BCL11A and ZBTB7A repressor binding regions, suggesting that activator binding at these positions may compete with repressor occupancy in addition to driving transcription.

#### 2.2.2 Cis‐regulatory architecture of the β‐globin locus

The chromatin architecture of the β‐globin locus is another key regulator of γ‐globin expression. Central to this is the β‐globin locus control region (LCR), a distal super‐enhancer upstream of the β‐globin gene cluster that interacts with globin gene promoters to drive developmentally appropriate transcription [62]. Studies of deletional HPFH, in which large deletions within the β‐globin locus lead to γ‐globin expression, support a model in which γ‐globin repression is mediated by competitive recruitment of the LCR to the *HBB* promoters. Analysis of genomic regions disrupted in deletional HPFH demonstrated that removal of a 13.6 kb genomic region encompassing the first intron of the *HBB* gene and adjacent upstream regulatory elements increases γ‐globin expression while reducing LCR interaction with the *HBB* promoter [63]. Subsequent studies refined this model by identifying a core region within the *HBB* promoter whose loss is sufficient to redirect LCR contacts toward the *HBG* promoters, establishing promoter competition as a key determinant of globin switching [64]. This redistribution of LCR contacts may be mediated, at least in part, by transcription factor occupancy at the *HBB* promoter. Disruption of the −78 TBP (TATA‐box binding protein) binding site reduces *HBB* promoter activity, increases γ‐globin expression, and enhances LCR interactions with the *HBG* promoters [53]. Together, these findings establish that transcription factor occupancy and promoter integrity at the *HBB* locus govern LCR allocation, thereby controlling the balance between adult and fetal globin gene expression.

Additional structural features within the β‐globin locus can also influence enhancer‐promoter interactions with the *HBG* genes. A single‐nucleotide resolution base‐editing screen of putative *HBG* regulatory elements identified functional regions within the non‐coding loci *BGLT3* and *HBBP1*, where sequence perturbations were associated with increased HbF expression [65]. Consistent with a structural role for this region, deletion of the *HBBP1* locus has been shown to increase γ‐globin expression and alter chromatin contacts to favor interactions between the LCR and the *HBG* promoters in CD34^+^ cells [66]. In addition, disruption of chromatin boundaries can also modulate enhancer access within the locus. Deletion of a CTCF (CCCTC‐binding factor) binding site downstream of *HBB* allows a distal enhancer located outside the β‐globin locus to interact with the *HBG* genes, leading to increased γ‐globin expression [67]. Collectively, these studies demonstrate that both intra‐locus regulatory elements and higher‐order chromatin boundaries shape enhancer accessibility, thereby modulating *HBG* expression.

#### 2.2.3 Trans‐acting genetic regulators of fetal hemoglobin

Recent studies combining human genetic analyses and functional base‐editing approaches have identified genetic variants that perturb trans‐acting regulators of *HBG* expression. These variants affect either the activity of epigenetic modifiers or the regulation of transcription factors, thereby modulating chromatin state and transcriptional repression at the *HBG* promoters. For example, introduction of a single‐nucleotide insertion −1368 bp upstream of the *HBG2* promoter creates a *de novo* binding site for the transcription factor FOXO3 (forkhead box O3), resulting in a marked increase in HbF expression [68]. This effect is accompanied by decreased enrichment of DNMT3A (DNA methyltransferase 3 alpha) at the *HBG* promoters, as well as reduced occupancy of the transcriptional repressors BCL11A and ZBTB7A, indicating that a single *de novo* transcription factor binding event can disrupt a coordinated repressive program at the *HBG* promoters. Similarly, a missense mutation in *DNMT1* identified in β‐thalassemia patients reduces its recruitment to the *HBG* promoters, impairing its interaction with BCL11A and leading to increased γ‐globin expression [69]. Additionally, a targeted adenine base‐editing screen of candidate variants enriched in individuals with elevated HbF identified regulatory elements controlling key trans‐acting factors, including the *BCL11A* enhancer, the *MYB‐HBS1L* intergenic region, and regions proximal to *KLF1* and *NFIX* [65]. Perturbation of these elements altered expression of their associated genes and increased γ‐globin levels, demonstrating that regulatory variants at these loci can directly modulate trans‐acting factor expression to influence *HBG* regulation.

### 2.3 Development of gene therapy approaches and technologies

HSPC gene editing has emerged as a central strategy for the treatment of SCD and β‐thalassemia. These approaches involve *ex vivo* modification of autologous CD34^+^ HSPCs, followed by reinfusion into patients. Recently, the FDA approved the first CRISPR/Cas9‐based gene editing therapy, CASGEVY (exagamglogene autotemcel), which disrupts the erythroid‐specific BCL11A enhancer to induce HbF expression. Clinical studies have demonstrated that this approach results in transfusion independence in β‐thalassemia and elimination of vaso‐occlusive crises in SCD [3, 70]. The lentiviral gene therapy LYFGENIA (lovotibeglogene autotemcel), which drives expression of an anti‐sickling β‐globin variant, has also demonstrated sustained production of therapeutic hemoglobin and reduction or elimination of vaso‐occlusive events in SCD [71].

Despite these advances, current therapeutic strategies incompletely address globin imbalance. HbF induction reduces the proportion of sickle hemoglobin but does not eliminate its production, and gene addition introduces functional β‐like globin without suppressing endogenous mutant β‐globin. These limitations have motivated the development of next‐generation gene editing strategies aimed at more comprehensive correction of globin composition. HUDEP‐2 and primary CD34^+^ cells have been widely used in the development and evaluation of these approaches. HUDEP‐2 cells enable rapid testing and comparison of novel gene therapy strategies, including multiplex perturbations and engineered constructs, whereas CD34^+^ HSPCs are required for preclinical evaluation, including assessment of editing efficiency, differentiation, and engraftment potential. The following studies demonstrate how these complementary systems have been leveraged to develop and refine gene editing therapies for β‐hemoglobinopathies.

#### 2.3.1 Novel gene editing approaches for β‐hemoglobinopathies

**Fig. 2:**
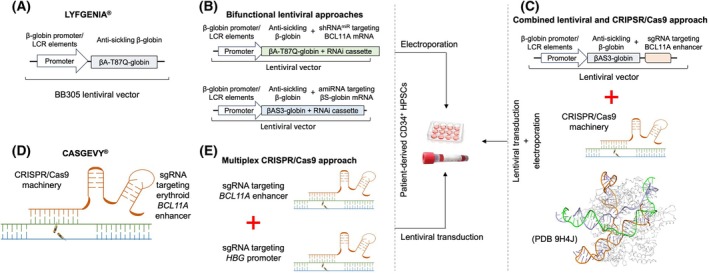
Summary of gene therapies to treat sickle cell disease (SCD) [3]. Graphics were made by using Microsoft PowerPoint version 16.109.3. (A) LYFGENIA is an FDA‐approved lentiviral gene therapy for SCD in which autologous CD34^+^ hematopoietic stem and progenitor cells are transduced *ex vivo* with the BB305 lentiviral vector encoding the anti‐sickling βA‐T87Q‐globin transgene. (B) Bifunctional lentiviral approaches combine anti‐sickling β‐globin gene addition with RNAi‐mediated suppression of disease‐relevant transcripts. One strategy, represented by the ATM1.1 vector, incorporates an RNAi cassette encoding a BCL11A‐targeting shRNA^miR^ into a βA‐T87Q‐globin lentiviral vector to promote concurrent production of modified adult hemoglobin and fetal hemoglobin. A second strategy, represented by βAS3m/miR7m, uses a modified βAS3‐globin lentiviral vector carrying an RNAi cassette encoding an amiRNA targeting endogenous βS‐globin mRNA, thereby reducing sickle β‐globin expression and favoring incorporation of βAS3‐globin into hemoglobin tetramers. (C) Combined lentiviral and CRISPR/Cas9 strategies pair LV‐mediated expression of an anti‐sickling β‐globin transgene with CRISPR/Cas9‐mediated editing of an additional HbF‐silencing regulatory target. This strategy is represented by LV.AS3mod.gR‐BCL11A, which encodes a modified βAS3‐globin transgene and an sgRNA targeting the BCL11A enhancer, with Cas9 delivered separately by electroporation. The approach is designed to increase production of both modified adult hemoglobin and fetal hemoglobin in patient‐derived CD34^+^ HSPCs. Structure of PDB 9H4J was generated by using the Schrödinger Open‐Source PyMol version 3.1.4. (D) CASGEVY is an FDA‐approved CRISPR/Cas9‐based gene editing therapy in which autologous CD34^+^ hematopoietic stem and progenitor cells (HSPCs) are edited *ex vivo* using an sgRNA targeting the erythroid‐specific BCL11A enhancer. Disruption of this enhancer reduces BCL11A expression during erythroid differentiation, thereby increasing *HBG1*/*HBG2* expression and fetal hemoglobin production. (E) Multiplex CRISPR/Cas9 approaches combine editing of the BCL11A erythroid enhancer with editing of regulatory sequences in the HBG1/HBG2 promoters, including BCL11A‐associated repressive motifs, to enhance fetal hemoglobin induction.

The efficacy of current gene therapy approaches depends on achieving sufficient levels of genetically modified HSPCs, with clinical outcomes correlating with the proportion of edited or transduced cells [3, 72]. This has motivated the development of strategies that increase therapeutic impact per modified cell, including combinatorial approaches that simultaneously target multiple components of globin regulation (Fig. 2).

The engineered β‐globin variants βT87Q (used in LYFGENIA) and βAS3 incorporate amino acid substitutions that reduce sickle hemoglobin polymerization [73] (Fig. 2A). The efficacy of gene addition strategies using these β‐globin variants can be further enhanced by combining anti‐sickle β‐globin expression with the perturbation of BCL11A, through knockdown (Fig. 2B) or CRISPR/Cas9‐mediated editing (Fig. 2C). These approaches enable concurrent production of HbF and modified adult hemoglobin, resulting in improved functional outcomes in CD34^+^ HSPCs and xenotransplant models [74, 75]. Similarly, suppression of endogenous sickle β‐globin in combination with anti‐sickling β‐globin expression improves functional outcomes in patient‐derived erythroid cells [76]. Complementary efforts have focused on extending the CASGEVY HbF induction strategy (Fig. 2D). Multiplex editing approaches that simultaneously target the BCL11A enhancer and HBG promoters result in greater HbF induction than single‐site editing (Fig. 2E) [77].

Strategies to enhance therapeutic efficacy of genetic therapies for β‐thalassemia have focused on directly correcting globin composition through targeted editing. In β‐thalassemia, excess unpaired α‐globin chains form toxic precipitates that impair erythroid maturation [78]. CRISPR/Cas9‐mediated reduction of α‐globin improves the α/β‐globin ratio and ameliorates disease‐associated phenotypes in patient‐derived CD34^+^ HSPCs and xenotransplant models [79]. In parallel, precise correction of disease‐causing point mutations using base editors restores endogenous β‐globin expression without introducing double‐strand DNA breaks, achieving high editing efficiencies across multiple β‐thalassemia variants [80]. Together, these approaches demonstrate that therapeutic efficacy can be enhanced through targeted modification of globin gene expression and composition.

#### 2.3.2 Novel gene editing technologies

Development of novel editing and vector technologies has largely been driven by practical limitations of existing platforms, including inefficient or toxic cargo delivery, restricted editing scope, and suboptimal vector architectures that reduce transgene expression or transduction efficiency. Recent studies have addressed these constraints through improved editor delivery methods, expanded editing chemistries, and erythroid‐specific vector elements that increase potency or specificity in erythroid cells.

Efficient delivery of gene editing machinery remains a central constraint in erythroid systems [81], particularly in primary HSPCs where viability and functional integrity must be preserved [82]. Some studies have focused on improving delivery through transient, non‐integrating modalities. For example, delivery of gene editing components as *in vitro* transcribed mRNA enables rapid, transient expression while avoiding the persistence and insertional risks associated with DNA‐based systems, resulting in high editing efficiencies in both HUDEP‐2 cells and CD34^+^ HSPCs [83]. In parallel, non‐viral nanoparticle‐based approaches have been developed to bypass electroporation‐associated toxicity. Poly(lactic‐co‐glycolic acid) (PLGA) nanoparticles encapsulating CRISPR‐Cas9 components support intracellular delivery and functional gene editing in erythroid cells while reducing delivery‐associated cytotoxicity relative to electroporation [84]. Together, these approaches highlight ongoing efforts to balance delivery efficiency, cellular viability, and scalability in therapeutically relevant erythroid systems.

Efficient intracellular cargo delivery to erythroid cells remains a distinct challenge due to their cytoskeletal architecture and limited endocytic activity during maturation. Systematic evaluation of multiple delivery platforms in erythroid precursor cells demonstrated that commonly used approaches, including several cell‐penetrating peptides and non‐specific uptake strategies, exhibit limited efficiency in this context. In contrast, delivery systems incorporating targeting mechanisms directed toward erythroid‐enriched surface receptors showed improved uptake and specificity, indicating that cell surface recognition, rather than cargo design alone, is a primary determinant of delivery efficiency in erythroid cells [85].

In parallel with delivery improvements, genome editing technologies have been refined to expand the scope and precision of sequence modification. Base editors enable targeted nucleotide substitutions without generating double‐strand breaks inherent to other genome editing technologies but were previously limited to single transition types. To address this constraint, dual base editors capable of mediating both cytosine and adenine conversions within a single system have been developed, enabling more flexible editing of genomic targets [86]. Complementary approaches have focused on improving the efficiency and reproducibility of editing workflows in erythroid model systems. Plasmid‐based CRISPR‐Cas9 editing combined with single‐stranded oligonucleotide donors supports efficient introduction of defined sequence changes in HUDEP‐2 cells and enables the rapid generation of stable, isogenic erythroid lines for functional studies [87].

In addition to delivery and editing strategies, effective gene therapy requires precise control of transgene expression within the erythroid lineage. Lineage‐restricted RNAi vectors have demonstrated that erythroid‐specific gene regulation can be achieved by placing short hairpin RNA expression under the control of β‐globin promoter and locus control region (LCR)‐derived regulatory elements, enabling selective knockdown of targets such as BCL11A and ZBTB7A while minimizing off‐target effects in non‐erythroid cells [88]. However, conventional globin gene therapy vectors that rely on the β‐globin LCR are constrained by its large size and complex architecture, which can limit vector design, packaging capacity, and transduction efficiency. Recent studies have addressed these limitations through the systematic identification of compact erythroid‐specific regulatory elements. High‐throughput screening of developmentally active regulatory sequences in HUDEP‐2 cells enabled the discovery of short enhancers that recapitulate or exceed LCR activity while maintaining lineage specificity [89]. Complementary approaches using lentiviral massively parallel reporter assays (LV‐MPRA) have refined the functional architecture of the LCR, enabling the design of optimized vectors with improved expression characteristics [90]. Together, these studies demonstrate that precise control of regulatory elements represents a critical component of next‐generation erythroid gene therapy strategies.

## 3 Cancer relevance of transcriptional and epigenetic regulators affecting HbF

**Fig. 3:**
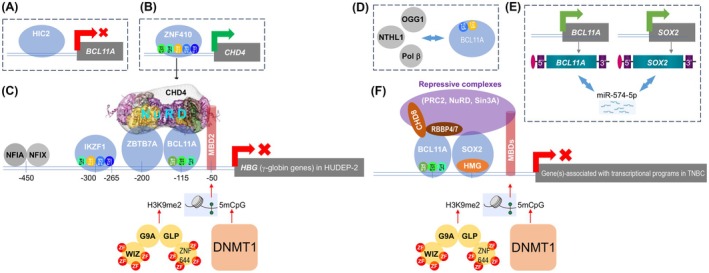
BCL11A‐mediated transcriptional and epigenetic programs in HUDEP‐2 cells (A‐C) and TNBC (D–F). Graphics were made by using Microsoft PowerPoint. (A) In human umbilical cord blood‐derived erythroid progenitor (HUDEP‐2) cells, HIC2 suppresses *BCL11A* expression. (B) ZNF410 regulates *CHD4* expression. (C) BCL11A, ZBTB7A, IKZF1, and NFIA/X bind individually or collaboratively to the promoters of γ‐globin genes (*HBG1* and *HBG2*), in association with the NuRD repressor complex, including CHD4 and MBD2 components. DNA methylation and histone H3 lysine 9 methylation act as epigenetic silencing marks. HIC2, ZNF410, IKZF1, ZBTB7A, BCL11A, WIZ and ZNF644 are C2H2 zinc finger proteins. (D) In triple‐negative breast cancer (TNBC), BCL11A promotes the activities of base‐excision repair enzymes, including OGG1, NTHL1 and DNA Polymerase β, thereby protecting cancer cells from oxidative DNA damage. (E) MicroRNA miR‐574‐5p binds to the mRNA of *BCL11A* and *SOX2*, reducing their protein levels. (F) BCL11A is linked to multiple chromatin‐associated mechanisms in TNBC, including DNMT1‐dependent repression of ISL1, interactions with RBBP4/7‐containing repressive complexes, and association with a CHD8 co‐regulatory complex. In parallel, the miR‐574‐5p/SOX2/BCL11A axis is implicated in SKIL/TAZ/CTGF‐driven proliferation, migration, and epithelial–mesenchymal transition (EMT). Additional DNA and histone methylation machinery shown, such as DNMT1 and MBD proteins, as well as H3K9me2 methyltransferase complex (G9A/GLP, WIZ, and ZNF644), represents broader epigenetic silencing pathways. BCL11A, B‐cell lymphoma/leukemia 11A; CHD4/8, chromodomain helicase DNA‐binding proteins 4 and 8; CTGF, Connective Tissue Growth Factor; DNMT1, DNA methyltransferase 1; GLP, G9a‐like protein or EHMT1, euchromatic histone methyltransferase 1; HIC2, hypermethylated in cancer 2; IKZF1, IKAROS family zinc finger 1; ISL1, ISL LIM homeobox 1; MBD, methyl‐CpG DNA‐binding domain. G9A (also known as EHMT2), euchromatic histone methyltransferase 2; NTHL1, NTH like DNA glycosylase 1; OGG1, 8‐oxoguanine DNA glycosylase; RBBP4/7, retinoblastoma binding proteins 4 and 7; SKIL, SKI‐Like Proto‐Oncogene; SOX2, SRY‐box transcription factor 2; TAZ, Transcriptional Coactivator with PDZ‐binding Motif; WIZ, widely interspaced zinc finger; ZBTB7A, zinc finger and BTB domain‐containing 7A; ZNF410, zinc finger protein 410; ZNF644, zinc finger protein 644.

Studies of γ‐globin regulation illustrate how transcription factors and chromatin‐associated proteins can impose stable gene expression states through coordinated DNA‐binding, recruitment of epigenetic modifiers, and control of cis‐regulatory element activity (Fig. 3A–3C). These same regulatory mechanisms are central to cancer biology, where malignant phenotypes are often sustained by aberrant transcriptional and chromatin states. Accordingly, many of the γ‐globin regulators discussed in this review also function in cancer‐associated regulatory networks, either by maintaining oncogenic transcriptional programs, repressing tumor suppressor or differentiation‐associated genes, or enabling adaptive phenotypes such as stemness, invasion, and therapy resistance.

Pharmacologic studies of HbF induction illustrate this connection by identifying small‐molecule strategies that perturb chromatin‐regulatory pathways relevant to both erythroid gene regulation and cancer therapy. DNMT1 inhibition represents the clearest point of overlap. Pharmacologic DNMT1 depletion or inhibition by 5‐azacytidine and decitabine increases HbF production in patients with SCD [91, 92] with early 5‐azacytidine studies linking this effect to hypomethylation of the β‐globin gene locus [93]. Azacitidine and decitabine are also established hypomethylating therapies for myeloid malignancies, with FDA‐approved use in myelodysplastic syndromes (MDS) and broader treatment use in acute myeloid leukemia‐related contexts [94, 95]. Newer non‐nucleoside DNMT1‐selective inhibitors further support this connection at the preclinical level. GSK3482364 induces γ‐globin and HbF expression in erythroid systems [96]. Related DNMT1‐selective inhibitors have also shown activity in cancer models, including GSK3685032, which induces DNA hypomethylation, transcriptional reactivation, cancer cell growth inhibition, and antitumor activity in AML models [97], and GSK‐3484862, which promotes rapid DNMT1 degradation and global hypomethylation in cancer cell lines [98]. How DNMT1‐dependent methylation contributes to cancer‐associated transcriptional programs is discussed in greater detail below. G9a/GLP inhibitors provide a second example of compounds that perturb chromatin‐regulatory pathways relevant to both HbF induction and cancer biology. In erythroid systems, UNC0638 induces HbF expression in adult human erythroblasts [99], while RK‐701 reactivates γ‐globin expression through a mechanism involving BGLT3 induction [56]. In cancer models, UNC0638 suppresses migration, invasion, tumorsphere formation, and anchorage‐independent growth in triple‐negative breast cancer (TNBC) cells [100]. Consistent with the cancer relevance of both DNMT and G9a/GLP targeting, dual inhibition of these epigenetic pathways has also shown activity in oncology models. The G9a/DNMT inhibitor CM‐272 inhibits proliferation, promotes apoptosis, and prolongs survival in acute myeloid leukemia, acute lymphoblastic leukemia (ALL), and diffuse large B‐cell lymphoma (DLBCL) xenograft models [101], while G9a/DNMT1 co‐targeting impairs malignancy and reduces tumor growth in non‐small cell lung cancer (NSCLC) models [102].

Together, these examples reinforce the broader theme that factors and pathways controlling fetal hemoglobin regulation can also participate in cancer‐associated transcriptional or epigenetic programs. Their broader involvement in cancer is summarized in Table 2, while the following sections emphasize mechanistic examples that directly connect regulatory function to malignant phenotype.

**Table 2:** Genes associated with γ‐globin repression and their potential roles in cancer from literature. ALL, acute leukocytic leukemia; AML, acute myeloid leukemia; BC, breast cancer; BCa, bladder cancer; CC, cerical carcinoma; ccRCC, clear cell renal cell carcinoma; CRC, colorectal cancer; EC, endometrial carcinoma; EOC, epithelial ovarian cancer; ESCC, esophageal squamous cell carcinoma; EwS, Ewing sarcoma; GC, gastric cancer; HCC, hepatocellular carcinoma; HNSCC, head and neck squamous cell carcinoma; GC, gastric cancer; GBM, glioblastoma; iCCA, Intrahepatic cholangiocarcinoma; LUSC, lung cell squamous carcinoma; LSCC, laryngeal squamous cell carcinoma; LUAD, lung adenocarcinoma; MCL, mantle cell lymphoma; MM, mesothelioma; NB, neuroblastoma; NET, neuroendocrine tumors; NKTL, natural killer/T‐cell lymphoma; OC, ovarian cancer; OS, osteosarcoma; PCa, prostate carcinoma; PDAC, pancreatic cancer; PTC, papillary thyroid carcinoma; RCC, renal cell carcinoma; RMS, rhabdomyosarcoma; SCLC, small cell lung cancer; TNBC, triple‐negative breast cancer; UBC, urothelial bladder cancer; UC, urothelial carcinoma; UM, uveal melanoma.

Gene (type)	NCBI ID	Role in cancer
BCL11A (B‐cell leukemia/lymphoma 11 A) Transcription factor	53335	Oncogene in AML [103, 104, 105] Oncogene in B‐cell lymphomas [106, 107, 108] Oncogene in NB [109, 110, 111] Prognostic marker/oncogenic association in NSCLC [112, 113, 114] Oncogene in LSCC [114, 115, 116] Oncogene in LUSC [113, 117] Oncogene in TNBC [118, 119, 120, 121, 122, 123]
ZBTB7A (zinc finger and BTB domain‐containing 7A) Transcription factor	51341	Tumor suppressor in AML [124, 125, 126] Oncogene in BC [127, 128, 129, 130] Oncogene in CRC [131, 132, 133] Oncogene in HCC [134, 135, 136, 137] Oncogene in NSCLC [138, 139, 140, 141] Oncogene in OC [142, 143] Oncogene in OS [144, 145] Tumor suppressor in PCa [146, 147]
HIC2 (hypermethylated in cancer 2) (BCL11A transcriptional repressor)	23119	Tumor suppressor in GBM [148, 149]
MEN1 (menin 1) Chromatin remodeler (MLL complex)	4221	Oncogene in pancreatic NET [37, 150, 151, 152] Oncogene in thymic NET [153, 154, 155] Oncogene in lung NET [156, 157, 158]
KMT2A (lysine methyltransferase 2A) (MLL complex)	4297	Oncogene in AML [159, 160, 161, 162] Oncogene in ALL [163, 164, 165]
ATF4 (activating transcription factor 4) Transcription factor	468	Oncogenic stress adaptation in BC [166, 167]
CHD4 (chromodomain helicase **D** NA binding protein 4) (NuRD complex)	1108	Oncogenic dependency in BC [168, 169, 170] Oncogene/oncogenic dependency in CRC [171, 172, 173] Tumor suppressor in EC [174, 175, 176] Oncogenic dependency in RMS [177, 178]
MBD2 (methyl‐CpG binding domain protein 2) (NuRD complex)	8932	Oncogenic dependency in CRC [179, 180]
HDAC2 (histone deacetylase 2) (NuRD complex)	3066	Oncogene/oncogenic dependency in CRC [181, 182, 183, 184] Oncogene in GC [185, 186] Oncogene in BC [187, 188, 189]
PRMT5 (protein arginine methyltransferase 5)	10 419	Oncogene in AML [190, 191, 192, 193] Oncogene in BC [194, 195, 196] Oncogene in BCa [197, 198, 199, 200] Oncogene in CRC [201, 202, 203, 204] Oncogene in ESCC [205, 206] Oncogene in GC [207, 208, 209] Oncogene in GBM [210, 211, 212, 213] Oncogene in HCC [214, 215, 216, 217] Oncogene in MCL [218, 219, 220, 221] Oncogene in NSCLC [222, 223, 224] Oncogene in OC [225, 226] Oncogene in PCa [227, 228, 229] Oncogene in PDAC [230, 231, 232, 233]
NFIA (nuclear factor I A) Transcription factor	4774	Context‐dependent role in GBM (evidence for oncogenic [234, 235, 236] and TSG [237, 238, 239] roles) Oncogene in ESCC [240, 241] TSG in NSCLC [242, 243]
NFIX (nuclear factor I X) Transcription factor	4784	Oncogene in GBM [244, 245, 246]
ERF (ETS2 repressor factor) Transcription factor	2077	TSG in PCa [247, 248]
MAZ (MYC‐associated zinc finger transcription factor)	4150	Oncogene in CRC [249, 250] Oncogenic dependency in GBM [251, 252] Oncogene in GC [253, 254] Oncogene in HCC [255, 256, 257, 258] Oncogene in PCa [259, 260] Oncogenic dependency in PDAC [261, 262] Oncogene in PTC [263, 264, 265] Oncogene in TNBC [266, 267]
BAP1 (BRCA1 associated protein 1) Deubiquitinase (DRED complex)	8314	TSG in BAP1‐inactivated melanocytic tumors (BIMTs) [268, 269, 270] TSG in MM [271, 272, 273, 274] TSG in UM [275, 276, 277, 278] TSG in ccRCC [279, 280, 281, 282] TSG in iCCA [283, 284, 285, 286] TSG in Meningioma (rhabdoid / BAP1‐altered subtype) [287, 288, 289]
DNMT1 (DNA methyltransferase 1)	1786	Oncogenic dependency in AML [97, 290, 291, 292] Oncogene in BCa [293, 294, 295, 296, 297] Oncogene in CC [298, 299, 300, 301] Oncogene in ccRCC [302, 303] Oncogene in CRC [304, 305, 306, 307, 308] Oncogene in EC [309, 310] Oncogene in EOC [311, 312, 313] Oncogene in ESCC [314, 315, 316, 317] Oncogene in GC [318, 319, 320] Oncogene in glioma [321, 322, 323] Oncogene in HCC [324, 325, 326, 327] Oncogene in HNSCC [328, 329, 330, 331] Oncogene in melanoma [332, 333, 334] Oncogene in NSCLC [102, 335, 336, 337] Oncogene in PDAC [338, 339, 340, 341] Oncogene in PCa [342, 343, 344, 345] Oncogene in UC [346, 347, 348]
KDM1A/LSD1 (lysine specific demethylase 1A)	23 028	Oncogenic dependency in AML [349, 350, 351, 352] Oncogene in CRC [353, 354, 355] Oncogene in EC [356, 357, 358] Oncogene in GBM [359, 360, 361] Oncogene in GC [362, 363, 364] Oncogene in HCC [365, 366, 367, 368] Oncogene in NB [369, 370, 371, 372] Oncogene in OC [373, 374, 375, 376] Oncogene in PCa [377, 378, 379, 380] Oncogene in PDAC [381, 382] Oncogenic dependency in SCLC [383, 384, 385, 386]
NR2C2/TR4 (nuclear receptor subfamily 2 group C member 2) Transcription factor	7182	TSG in HCC [387, 388] Oncogene in PCa [389, 390, 391, 392] Oncogene in RCC [393, 394]
SPOP (speckle‐type BTB/POZ protein) Substrate adaptor (Cul3‐based E3 ubiquitin ligase complex)	8405	Oncogene in ccRCC [395, 396, 397, 398, 399] Tumor suppressor in EC [174, 400, 401] Tumor suppressor in PCa [402, 403, 404, 405, 406]

### 3.1 Epigenetic regulators and malignant cell‐state maintenance

The NuRD ATPase CHD4 provides a particularly clear example of how chromatin remodeling can support malignant transcriptional states. In fusion‐positive rhabdomyosarcoma, CHD4‐containing NuRD complexes localize to super‐enhancers and maintain chromatin accessibility required for binding of the PAX3 (paired box 3)–FOXO1 (forkhead box O1) fusion oncoprotein, thereby supporting RNA polymerase II positioning, oncogenic transcription, and tumor cell survival [177]. This study also identified CHD4 as a broad cancer dependency in genome‐wide vulnerability datasets, although the super‐enhancer mechanism was defined specifically in fusion‐positive rhabdomyosarcoma. In colorectal cancer, CHD4 supports malignancy through a distinct repressive mechanism, promoting DNA methyltransferase recruitment, *de novo* DNA methylation, and maintenance of hypermethylation‐associated silencing of tumor suppressor genes [171].

DNA methylation provides another mechanism for enforcing malignant cell states. DNMT1 is the major maintenance DNA methyltransferase, and cancer cells can depend on DNMT1 activity to preserve methylation‐associated repression of tumor suppressor and differentiation‐associated programs. In acute myeloid leukemia, DNMT1‐dependent methylation contributes to leukemic maintenance by silencing tumor suppressor programs. Increased DNMT1 expression is associated with promoter methylation and reduced expression of CDH1 (cadherin 1), PTEN (phosphatase and tensin homolog), and BRCA1 (BRCA1 DNA repair associated) [290], while reduced DNMT1 expression delays leukemogenesis and impairs leukemia stem cell self‐renewal through derepression of tumor suppressors [407]. Similar methylation‐dependent tumor suppressor repression has also been reported in solid tumors, where DNMT1 knockdown demethylates and reactivates genes such as RASSF1A (Ras association domain family member 1A) and APC (APC regulator of Wnt signaling pathway) in lung cancer [408], or RASSF1A and DAPK (death associated protein kinase 1) in esophageal squamous cell carcinoma (ESCC) with accompanying suppression of tumor growth and invasive phenotypes [314]. The clinical use of hypomethylating agents in myeloid malignancies further supports the therapeutic relevance of DNA methylation pathways to cancer progression, although these effects are mechanistically complex and may include additional mechanisms beyond DNMT1‐dependent hypomethylation [409].

Similarly, the chromatin‐associated histone demethylase KDM1A/LSD1 promotes leukemic cell states by sustaining transcriptional repression at enhancer‐regulated differentiation programs. In AML and erythroleukemia models, LSD1 occupies GFI1 (growth factor independent 1 transcriptional repressor)/GFI1B (growth factor independent 1B transcriptional repressor)‐associated repressive complexes at enhancers, including the GFI1 super‐enhancer, thereby preserving an undifferentiated leukemic state. Disruption of this axis, either by pharmacologic KDM1A/LSD1 inhibition or CRISPR‐mediated deletion of the GFI1 super‐enhancer, releases enhancer repression, activates differentiation‐associated transcriptional programs, and promotes or modulates leukemic cell differentiation [349, 410].

### 3.2 Transcription factors in cancer‐associated regulatory networks

BCL11A is the clearest example of a transcription factor discussed in this review whose therapeutic relevance in hemoglobinopathies is paralleled by mechanistic cancer evidence (Fig. 3D–3F). In TNBC, BCL11A is recurrently amplified or overexpressed and supports tumor initiation, mammary stem/progenitor cell activity, and tumor maintenance [118]. Mechanistically, BCL11A promotes breast cancer stemness and tumor progression through activation of Wnt/β‐catenin signaling [119], while miR‐137‐mediated suppression of BCL11A reduces TNBC stemness and tumorigenesis by disrupting a BCL11A‐DNMT1 interaction that regulates ISL1 (ISL LIM homeobox 1) expression [120]. BCL11A also engages chromatin‐regulatory complexes in TNBC. It interacts with RBBP4/7 (RB binding protein 4/7 chromatin remodeling factor), shared subunits of PRC2 (polycomb repressive complex 2), NuRD, and SIN3A (SIN3 transcription regulator family member A)‐associated repressive complexes, and disruption of this interaction reduces breast cancer stem cell phenotypes [411]. More recent work identified CHD8 (chromodomain helicase DNA‐binding protein 8) as a TNBC‐specific BCL11A interaction partner that co‐regulates oncogenic transcriptional programs [121]. In parallel, miR‐574‐5p has been reported to suppress TNBC progression by targeting both BCL11A and SOX2 (SRY‐box transcription factor 2), thereby inhibiting the SKIL (SKI‐like proto‐oncogene)‐TAZ/WWTR1 (WW domain‐containing transcription regulator 1)‐CTGF/CCN2 (cellular communication network factor 2) axis [412]. Beyond these transcriptional and chromatin‐associated mechanisms, BCL11A also supports TNBC cell fitness through a noncanonical role in base‐excision repair, interacting with repair factors including OGG1 (8‐oxoguanine DNA glycosylase), NTHL1 (nth like DNA glycosylase 1), and Pol β to limit oxidative DNA damage and senescence [122, 413]. Together, these studies indicate that BCL11A functions in TNBC through multiple mechanisms that converge on tumor cell‐state maintenance, stemness, and survival.

In hematologic malignancies, developmental transcription factors can also shape malignant cell states by preserving or disrupting differentiation‐associated regulatory programs. ZBTB7A illustrates how loss of a differentiation‐regulatory transcription factor can cooperate with oncogenic fusion programs. In t(8;21) AML [400], recurrent ZBTB7A mutations disrupt DNA‐binding/repressor function, and ZBTB7A loss perturbs myeloid differentiation, increases glycolytic capacity, and permits RUNX1 (RUNX family transcription factor 1)–RUNX1T1 (RUNX1 partner transcriptional corepressor 1) dependent clonal expansion of human CD34^+^ cells [124, 125]. MYB provides a complementary example, in which AML cells retain dependence on a developmental transcription factor through assembly of MYB–CBP (encoded by CREB binding lysine acetyltransferase, *CREBBP*/P300 (EP300 lysine acetyltransferase)) coactivator complexes at leukemogenic regulatory elements. Disruption of the MYB–p300 interaction prevents transformation by multiple AML oncogenes, supporting a functional requirement for this coactivator interface during leukemogenesis [414]. Consistent with this model, peptidomimetic blockade of MYB–CBP/P300 disrupts complex assembly in AML cells, displaces MYB from oncogenic enhancers, and reduces MYB‐dependent expression of leukemogenic targets including BCL2 (BCL2 apoptosis regulator) and MYC (MYC proto‐oncogene, bHLH transcription factor) [415, 416]. Together, these examples show that hematopoietic transcription factors contribute to AML either by losing differentiation‐regulatory functions that restrain transformation or by maintaining enhancer‐associated leukemic survival programs.

In other contexts, transcription factors contribute to malignancy by reshaping broader regulatory networks rather than by controlling a single lineage program. In prostate cancer, ERF functions as a tumor‐suppressive ETS (E26 transformation‐specific) factor that restrains androgen receptor (AR)‐associated transcriptional output. Loss of ERF expands AR chromatin occupancy and transcriptional activity in PTEN‐deficient prostate models, producing a regulatory state that overlaps with the oncogenic effects of ERG (ETS transcription factor ERG), another ETS factor recurrently activated in prostate cancer [247, 417]. This supports a mechanism in which altered balance among ETS family transcription factors redirects AR‐dependent regulatory networks toward tumorigenesis. AFT4 engages in a distinct form of network‐level regulation, in which activation of the integrated stress response directly promotes transcriptional programs that allow cancer cells to tolerate hostile microenvironment conditions. Under nutrient deprivation, GCN2 (encoded by eukaryotic translation initiation factor 2 alpha kinase 4, *EIF2AK4*)‐ATF4 signaling induces amino acid metabolism genes including ASNS [asparagine synthetase (glutamine‐hydrolyzing)] to support tumor cell survival and proliferation [418], while ATF4 promotes TNBC aggressiveness through TGFβ (transforming growth factor beta 1)‐associated SMAD2/3/4 (SMAD family member 2/3/4) and mTORC2 (mechanistic target of rapamycin complex 2) signaling [166] and induces HMOX1/HO‐1 (heme oxygenase‐1) to promote anoikis resistance and metastatic behavior [419]. Together, these examples show that the transcription factors discussed here can promote cancer through higher‐order regulatory mechanisms, including redirection of hormone‐responsive transcriptional networks and activation of stress‐adaptive survival programs.

Selected transcription factors also promote cancer progression by activating gene programs that drive migration, invasion, and metastasis. In glioblastoma (GBM), NFIX directly activates EZR (ezrin), linking transcriptional regulation to cytoskeletal remodeling and cell motility; the identification of an NFIX response element within the EZR promoter, together with rescue of NFIX‐depletion‐induced migration defects by Ezrin restoration, supports a direct NFIX–EZR mechanism controlling GBM migration [244]. MAZ drives related invasion‐associated programs in epithelial malignancies. In hepatocellular carcinoma (HCC), MAZ promotes invasion and metastasis through induction of EMT‐associated regulators including ZEB1 and ZEB2 (zinc finger E‐box binding homeobox 1 and 2) [255], while in pancreatic ductal adenocarcinoma (PDAC), MAZ acts downstream of Cyr61/CCN1 (cellular communication network factor 1) to promote invasion through CRAF (encoded by Raf‐1 proto‐oncogene, serine/threonine kinase; *RAF1*)–ERK signaling [261]. TR4/NR2C2 provides an additional example in prostate cancer, where TR4 promotes invasion of CD133^+^ prostate cancer stem/progenitor‐like cells by increasing EZH2 (enhancer of zeste 2 polycomb repressive complex 2 subunit) expression and activating metastasis‐associated genes including NOTCH1 (notch receptor 1), SLUG (also referred to as snail family transcriptional repressor 2; *SNAI2*), TGFβ1 (transforming growth factor beta 1), and MMP9 (matrix metallopeptidase 9) [389].

### 3.3 Gene editing and gene‐regulatory therapies in cancer

Therapeutic targeting of cis‐regulatory elements offers one route to disrupt oncogenic transcriptional dependencies in cancer. The clinical success of BCL11A enhancer editing in β‐hemoglobinopathies provides a useful conceptual precedent: disease‐relevant transcriptional states can sometimes be altered by targeting the regulatory elements that control them, rather than by directly targeting the encoded protein. In cancer, this logic is most compelling when a defined enhancer or chromatin dependency can be linked to an oncogenic transcriptional program. In T‐cell acute lymphoblastic leukemia (T‐ALL), for example, a somatic noncoding insertion upstream of TAL1 (TAL bHLH transcription factor 1, erythroid differentiation factor) creates a MYB‐bound oncogenic super‐enhancer that drives TAL1 expression. Genetic deletion of this mutant enhancer, as well as CRISPR‐based epigenetic repression of enhancer activity, reduces TAL1 expression and impairs leukemic cell growth, including in xenotransplant models [420, 421]. This provides a direct cancer parallel to regulatory element targeting, in which perturbation of a noncoding element disrupts a transcription factor‐driven malignant phenotype.

Programmable promoter and epigenome editing extend this strategy beyond enhancer disruption by enabling selective repression or correction of cancer‐relevant regulatory states. Mutations in the TERT (telomerase reverse transcriptase) promoter result in aberrant transcription factor binding and telomerase activation in multiple cancers. CRISPR interference or base‐editing approaches targeting mutant TERT promoter alleles suppress TERT expression, reduce telomerase activity, and inhibit tumor growth in preclinical glioblastoma models [422, 423, 424]. Epigenome editing may also be used to alter therapy‐response programs without permanently modifying DNA sequence. In glioblastoma, transient delivery of CRISPRoff targeted to the MGMT (O6‐methylguanine DNA methyltransferase) promoter produced durable MGMT silencing and sensitized tumor cells, including patient‐derived cultures and orthotopic xenografts, to temozolomide [425]. This example extends the logic of regulatory element targeting beyond oncogene suppression, showing that programmable epigenetic repression can also be used to modify clinically relevant resistance mechanisms.

Together, these studies suggest that the regulatory logic established through hemoglobinopathy gene therapy—the precise perturbation of disease‐relevant transcriptional control—can be extended to cancer, although most cancer applications remain preclinical and will require solutions for delivery, specificity, tumor heterogeneity, and reversibility. At present, targeted degradation of transcriptional and chromatin‐associated dependencies, exemplified by BET/BRD4 (bromodomain‐containing protein 4) degraders that suppress MYC‐associated transcriptional programs, offers a pharmacologic route to perturb oncogenic regulatory circuits when direct genome or epigenome editing is not yet feasible [426].

## 4 Conclusions

Fetal hemoglobin regulation provides a powerful example of how detailed mechanistic dissection of transcriptional control can lead to therapeutic intervention. Work in HUDEP‐2 cells and complementary erythroid systems has defined key regulators of γ‐globin silencing, including BCL11A, ZBTB7A, NuRD‐associated proteins, DNA methylation pathways, and cis‐regulatory elements within and beyond the β‐globin locus. These discoveries have informed gene‐regulatory therapies for β‐hemoglobinopathies and demonstrate that stable disease‐associated transcriptional states can be therapeutically reprogrammed. The relevance of these findings to oncology is not based on a direct equivalence between hemoglobinopathies and cancer, but rather on shared regulatory logic. Malignant cell states are often maintained by aberrant transcription factor activity, chromatin remodeling, enhancer‐promoter architecture, and epigenetic repression, and several HbF regulators discussed in this review have context‐dependent oncogenic or tumor‐suppressive functions. Thus, studies of HbF regulation offer a focused model for identifying regulatory dependencies, validating their functional consequences, and developing strategies to perturb them with precision. Future work that integrates mechanistic perturbation in tractable systems with disease‐relevant cancer models may extend these principles toward therapeutic targeting of transcriptional circuitry in oncology.

## Conflict of interest

The authors declare no conflict of interest.

## Author contributions

MY performed the literature review, conceptualized the manuscript, wrote the original draft, and led manuscript revision and editing. PD and XZ participated in discussion. XC is a Cancer Prevention and Research Institute of Texas (CPRIT) scholar in cancer research and participated in review and editing the manuscript.
